# Genetic differentiation and intrinsic genomic features explain variation in recombination hotspots among cocoa tree populations

**DOI:** 10.1186/s12864-020-6746-2

**Published:** 2020-04-29

**Authors:** Enrique J. Schwarzkopf, Juan C. Motamayor, Omar E. Cornejo

**Affiliations:** 1 School of Biological Sciences, Washington State University, Pullman, WA USA; 2 Universal Genetic Solutions, LLC, Miami, USA

**Keywords:** Recombination, Recombination hotspots, Domestication

## Abstract

**Background:**

Recombination plays an important evolutionary role by breaking up haplotypes and shuffling genetic variation. This process impacts the ability of selection to eliminate deleterious mutations or increase the frequency of beneficial mutations in a population. To understand the role of recombination generating and maintaining haplotypic variation in a population, we can construct fine-scale recombination maps. Such maps have been used to study a variety of model organisms and proven to be informative of how selection and demographics shape species-wide variation. Here we present a fine-scale recombination map for ten populations of *Theobroma cacao* – a non-model, long-lived, woody crop. We use this map to elucidate the dynamics of recombination rates in distinct populations of the same species, one of which is domesticated.

**Results:**

Mean recombination rates in range between 2.5 and 8.6 cM/Mb for most populations of *T. cacao* with the exception of the domesticated Criollo (525 cM/Mb) and Guianna, a more recently established population (46.5 cM/Mb). We found little overlap in the location of hotspots of recombination across populations. We also found that hotspot regions contained fewer known retroelement sequences than expected and were overrepresented near transcription start and termination sites. We find mutations in FIGL-1, a protein shown to downregulate cross-over frequency in Arabidopsis, statistically associated to higher recombination rates in domesticated Criollo.

**Conclusions:**

We generated fine-scale recombination maps for ten populations of *Theobroma cacao* and used them to understand what processes are associated with population-level variation in this species. Our results provide support to the hypothesis of increased recombination rates in domesticated plants (Criollo population). We propose a testable mechanistic hypothesis for the change in recombination rate in domesticated populations in the form of mutations to a previously identified recombination-suppressing protein. Finally, we establish a number of possible correlates of recombination hotspots that help explain general patterns of recombination in this species.

## Background

Genetic recombination is an important source of genome-wide genetic variation fundamental for evolutionary forces like selection and genetic drift to act. Selection and drift contribute to a loss of variation, which means that in the absence of forces that maintain variation along the genome, populations would be incapable of evolving over prolonged periods of time. Recombination rearranges genetic material onto different backgrounds generating a larger set of allele combinations on which selection can act. This rearrangement allows for more efficient selection, preventing mutations at different sites from affecting each other’s eventual fate (i.e. reducing Hill-Robertson interference) [1]. Different regimes of recombination can strongly influence how efficient selection is at purging deleterious mutations and increasing the frequency of beneficial mutations in the population [1].

Studies in a wide range of species have shown that recombination rates are not uniform along the genome and general patterns of variation have been described [2–12]. One pattern that has been observed in multiple species is the reduced recombination rate in centromeric regions of the chromosomes and the progressive increase of recombination rates as the physical distance from the telomeres decreases [2–5, 9, 10]. This pattern has also been shown to arise in simulation studies [13]. Another interesting pattern that has been observed is that of regions with unusually high rates of recombination spread throughout chromosomes: recombination hotspots [6, 12, 14–17]. The importance of recombination hotspots lies in their ability to shuffle genetic variation at higher rates than the rest of the genome, profoundly impacting the dynamics of selection for or against specific mutations [1]. In this study, we focus on locally defined recombination hotspots, requiring that their recombination rate be unusually high when compared to neighboring regions.

A variety of genomic features have been identified as being associated with regions of high recombination. Recombination hotspots have been linked to transcriptional start sites (TSSs) and transcriptional termination sites (TTSs) in *Arabidopsis thaliana*, *Taeniopygia guttata*, *Poephila acuticauda*, and humans [18–20]. In *Mimulus guttatus* hotspots were found to be associated with cpg islands (short segments of cytosine and guanine rich DNA, associated with promoter regions) [15]. CpG islands were also associated with increased recombination rates in humans and chimpanzees [21]. These patterns point to recombination occurring frequently near, but not within, coding regions. The formation of chiasmata is important for the proper disjunction of chromosomes during meiosis [22], but repeated double-strand breaks can lead to an increased mutation rate [23]. In coding regions in particular, this excess mutation rate can have a high evolutionary cost, due to the likelihood of novel deleterious mutations being higher than that of beneficial ones [24–28]. Recombination hotspots have also been found to be correlated with particular DNA sequence motifs. In some mammals, including *Mus musculus* [14] and apes [16, 21] binding sites for PRDM9, a histone trimethylase with a DNA zinc-finger binding domain, have been found to correlate with recombination hotspots. In *A. thaliana*, proteins that limit overall recombination rate have been identified, leading to a genome-wide increase in recombination rate in knockout mutants [29]. However, these *Arabidopsis* proteins have not been shown to direct recombination to particular regions and are therefore not expected to affect the location of recombination hotspots.

The dynamics of recombination hotspots shared between related species or populations of the same species have been investigated in apes, yielding varying results. Hinch et al. (2011) found that, at finer scales, the genetic maps of European and African human populations were significantly different [30]. They also found that, when looking at hotspots in the major histocompatibility complex, the African populations showed a hotspot that was not present in Europeans, but all European hotspots were found in African populations [30]. Recent work on recombination in apes found little correlation of recombination rates in orthologous hotspot regions when looking between species, but a strong correlation when comparing between two populations of the same species [16]. Other studies have also found very little sharing of hotspots between humans and chimpanzees [31, 32]. Additionally, the dynamic of changing hotspot locations observed in humans and other apes has been observed in simulations [33]. The disparity of empirical results regarding hotspots shared between related populations suggest that further work is required to disentangle the relationship between demographics and shared hotspots.

The identification of ten genetically differentiated populations of the cocoa tree, *Theobroma cacao*, [34, 35] can be leveraged to study population-level dynamics of recombination patterns. The ten *T. cacao* populations originate from different regions of South and Central America, and include one fully domesticated population (Criollo), used in the production of fine chocolate, and nine wilder, more resilient populations which generate higher cocoa yield than the Criollo variety (Fig. S1, Table S5) [34–36]. These ten populations have been shown to have strong signatures of differentiation between them (F_ST_ values ranging from 0.16 to 0.65, Table S6) and they separate into clear clusters of ancestry [35]. *T. cacao* has a mix of self-incompatible and self-compatible mating strategies. The proportion of self-fertilization greatly varies across populations with Criollo and Amelonado being the populations presenting higher levels of selfing and Iquitos and Nacional presenting lower frequency of self-fertilization (see Figure 5 in [35]). During domestication, recombination plays an important role in the segregation of traits, and for this reason it has been hypothesized that recombination rates will increase during the process of domestication [37]. Domestication can be a rapid process and there is theoretical evidence for the increase of recombination rates during periods of rapid evolutionary change [38]. Empirical evidence for this prediction has been shown in a limited number of herbaceous plant species with short generation times [39]. It is not clear if plant species with longer generation times are also expected to experience increased recombination rates, and it is also unclear what mechanisms could explain these differences. One possible explanation for differences in recombination rates between wild and domesticated populations is polymorphism in genes like those previously demonstrated to suppress recombination in *Arabidopsis thaliana* [40, 41]. The differences in recombination rates between wild and domesticated populations is just one of the possible questions that can be touched on with this system.

The ten populations of *T. cacao* also allow us to compare the locations of hotspots between them, potentially contributing to the understanding of hotspot turnover at the population-divergence timescale. These comparisons can also contribute to our understanding of how demographics impact the turnover of recombination hotspot locations. *T. cacao* is unique in this case for being a long-lived organism with no known driver of recombination hotspots (e.g. PRDM9). What contributes to the location of recombination hotspots in such a species is, of course, contingent on our being able to detect recombination hotspots in the different populations of *T. cacao*.

In order to locate recombination hotspots for *T. cacao* populations, we must first obtain fine-scale recombination maps for each population, which we did using an LD-based method. Fine-scale, LD-based recombination maps have been constructed for a number of plant models [12, 15, 19], identifying a variety of features correlated to recombination rate. Unlike these model plants with short generation times, *T. cacao* is a perennial woody plant with a five-year generation time [36]. The size and long generation time of *T. cacao* makes direct measurements of recombination impractical. However, historical recombination can be estimated for *T. cacao* using coalescent based methods [42]. Theoretical studies have shown that population structure can generate artificially inflated measures of LD [43, 44], which would be detrimental to our estimates of recombination. For this reason, recombination maps were constructed independently for each population. For each population we aim to describe the relationship between recombination hotspots and a variety of evolutionary and genomic factors.

We used an LD-based method to estimate recombination rates for ten populations of *T. cacao*, which we then analyzed with a maximum likelihood statistical framework to infer the location of recombination hotspots. The locations of hotspots were compared across populations and a novel resampling scheme tailored to the genomic architecture of *T. cacao* was used to generate null assumptions for the distribution of hotspots along the genome. These null distributions were used to identify differential representation of known DNA sequence motifs in ubiquitous recombination hotspots, and of overlap between recombination hotspots and genomic traits for each population. The re-sampling schemes used to identify these associations are novel in the context of this work and were designed to take into account the size and distribution of elements in the genome. In this work we aimed to answer the following questions: (i) How are recombination rates distributed within 10 highly differentiated populations of *T. cacao*, and how do they compare to each other? (ii) How are hotspots distributed along the genome of each of the ten populations of *T. cacao*, and can these distributions be explained by patterns of population genetic differentiation? (iii) Are there identifiable DNA sequence motifs that are associated with the location of recombination hotspots along the *T. cacao* genome? (iv) Are there genomic features (e.g. TSSs, TTSs, exons, introns) consistently associated with recombination hotspot locations across *T. cacao* populations? Our findings suggest that recombination hotspot locations generally follow patterns of diversification between populations, while also having a strong tendency to occur close to TSSs and TTSs. Moreover, we find a strong negative association between the occurrence of recombination hotspots and the presence of retroelements.

## Results

### Comparing recombination rates between populations

**Fig. 1:**
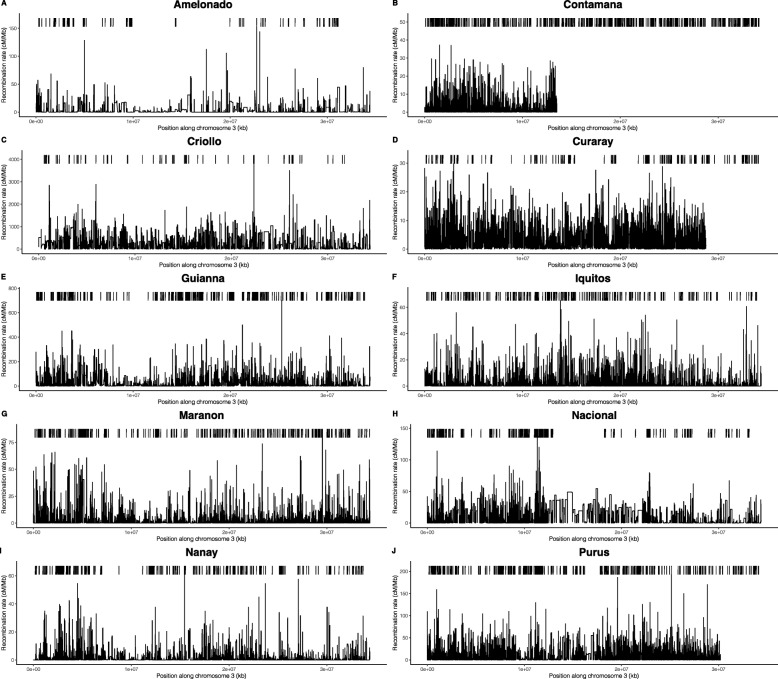
Recombination rates (*r*, in cM/Mb*)* and recombination hotspot locations (bars above the rates) fo the third chromosomes of all ten *T. cacao* populations: (**a**) Amelonado, (**b**) Contamana, (**c**) Criollo, (**d**) Curaray, (**e**) Guianna, (**f**) Iquitos, (**g**) Maranon, (**h**) Nanay, and (**j**) Purus . Maps of all chromosomes of all populations can be found at the github repository *(*https://github.com/ejschwarzkopf/recombination-map*)*

Populations show a mean recombination rate (*r*) between 2.1 and 525 cM/Mb (Table 1), with a variety of distributions (Fig. S2). We observe a higher mean than median *r* indicating that extreme high values are present for all populations. The extreme recombination rate values affect the mean, driving it to values consistently higher than the median. The pattern of recombination rates along the genome varied between populations, as can be seen in the comparison of the Nanay and Purus third chromosome (Fig. 1). Purus appears to have a higher average recombination rate than Nanay for chromosome three. More specifically, particular regions of the chromosome present peaks in one population that are absent in the other. A similar pattern can also be observed for the density of recombination hotspots, e.g. Purus presenting a high density of hotspots in certain regions that is not observed in Nanay. The median 95% probability interval for recombination rate across the genome for each population was found to be several orders of magnitude larger than the uncertainty per site, estimated as the median 95% Credibility Interval of the trace for each position in the genome for that population (Table S1).

**Table 1:** Recombination rates in *ρ = 4Ner* (in Morgans per base) and *r* (in cM/Mb) for all ten *T. cacao* populations. The *Ne* (from [35]) used to transform *ρ* to *r* for each population is also reported, as are the lower and upper bounds of a 95% confidence interval for mean *r*

Population	Mean *4N*_*e*_*r*	Mean *N*_*e*_	Mean *r* (cM/Mb)	Median *r* (cM/Mb)	L bound (Mean *r*)	U bound (Mean *r*)
Amelonado	1.58e-03	15,744	2.51	2.40e-04	2.48	2.54
Contamana	8.53e-03	61,102	3.49	4.92e-01	3.48	3.50
Criollo	1.46e-04	695	525	427	523	527
Curaray	1.04e-04	58,213	4.45	1.78	4.44	4.46
Guianna	8.66e-03	4651	46.5	7.74e-01	46.3	46.7
Iquitos	4.23e-03	49,984	2.11	5.88e-04	2.10	2.12
Maranon	4.09e-03	34,037	3.01	1.64e-03	2.99	3.02
Nacional	4.66e-03	26,060	4.47	9.76e-03	4.44	4.49
Nanay	6.82e-03	42,429	4.02	1.51e-02	4.00	4.04
Purus	5.95e-03	17,357	8.57	7.74e-01	8.54	8.60

Overall, the mean recombination rate for most of the populations is similar to that found for *Arabidopsis thaliana* using LDhat, when using θ = 0.1 [19] (Table 1). The LDhat estimates using θ = 0.001 were slightly higher than the estimates using θ = 0.1 for each population. We chose to proceed with analyses using the results from the θ = 0.1 since more of the mean population recombination rates fell within the range of values identified in plants [45] (Table S2).

In order to compare the median recombination rates of the different populations, we conducted a Kruskal-Wallis test (*p* < 2.2e-16) and Wilcoxon rank-sum tests for every pair of populations. The only pair that did not show a significant difference in median recombination rate was that of Nacional and Nanay (*p = 0.3*). All other pairwise comparisons were highly significant (*p < 2e-16*). Two populations, Guianna and Criollo, have a higher average recombination rate than the other populations by one and two orders of magnitude respectively (Table 1). Guianna and Criollo also have been estimated to have a lower effective population size (*N*_*e*_) [35] by one and two orders of magnitude respectively. However, there was no significant association between mean *N*_*e*_ and mean or medain *r* (mean *r: p = 0.1119*, median *r: p = 0.1482*), indicating that, for a high enough *N*_*e*_, the ability to detect recombination events is not dictated by the effective population size. When Criollo and Guianna were excluded, the relationships were also absent (mean *r: p = 0.3886*, median *r: p = 0.335*). When all populations were included, the inbreeding coefficient (*F*, from [35]) showed no significant linear association with mean or median *r* (mean *r: p = 0.336*, median *r: p = 0.381*). We also found no linear trend between sample size and mean or median *r* (mean *r: p = 0.233*, median *r: p = 0.228*).

**Fig. 2:**
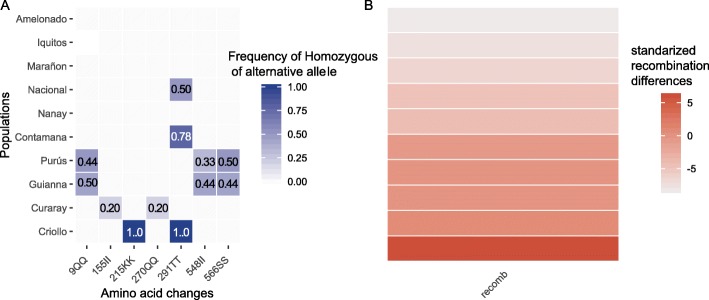
**a** frequency of individuals that are homozygous for the alternative allele of amino acid mutations in a *T. cacao* FIGL-1 ortholog. The alternative allele is defined in terms of the Amelonado reference genome. Blank squares have a frequency of zero. **b** *log*_*e*_ transformed recombination differences. The populations are in the same order in both panels

The FIGL-1 and FLIP proteins characterized by Fernandes et al. (2018a) were found to be responsible for recombination suppression in *Arabidopsis* [29]. Plants with a FIGL-1 knockout were found to increase recombination rates significantly and FLIP knockouts show increases of recombination at a much lesser extent [41]. Therefore, we explored the possibility that missense FIGL-1 and FLIP orthologs in *T. cacao* explain the between-population differences in recombination rate. We used a reciprocal BLAST search to identify the orthologs for both genes and used annotation data from Cornejo et al. (2018) to identify 15 missense mutations in FIGL-1 and 18 missense mutations in FLIP (Fig. 2, Fig. S3, Table S3) [35]. We then used a generalized linear model framework to infer the impact of the 8 uncorrelated missense mutations in the *T. cacao* FIGL-1 ortholog under the assumption of a full recessive model. We find that mutations 215KK (Coeff. = 426.54, *p* < 0.001), 155II (Coeff. = 8.97, p < 0.001), and 291TT (Coeff. = 0.47, *p* = 0.047) significantly explain changes in the recombination rate, but all other mutations made no significant impact. The same model was run for FLIP but returned no significant coefficients (after eliminating perfectly correlated mutations with those found in FIGL-1).

### Comparing recombination hotspot locations between populations

**Fig. 3:**
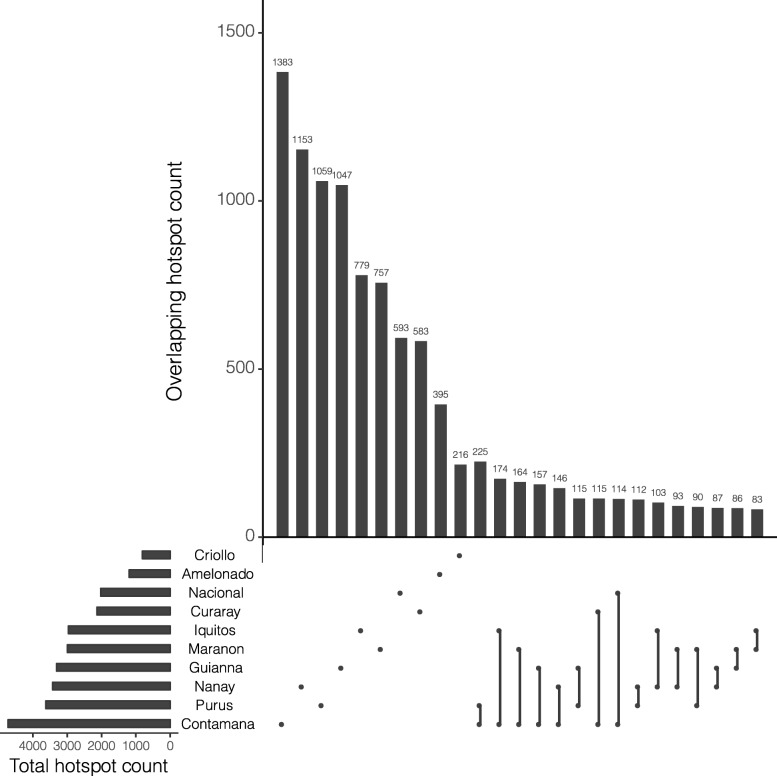
Upset plot showing number of hotspots in different subsets. Horizontal bars represent total hotspots detected in a population, each dot on the matrix indicate that the vertical bar above it is the count of hotspots unique to that population, connected dots indicate that the vertical bar above them represents hotspots shared between the populations represented by the connected dots. The 25 most numerous sets of hotspots are shown

The majority (55.5%) of hotspots identified were not shared between populations. The 25 most numerous sets of hotspots are represented in Fig. 3. The nine largest of these are sets of hotspots unique to single populations. The hotspots unique to the remaining population (Criollo) formed the eleventh largest set. Effective population size (*N*_*e*_) is not a good linear predictor of the number of detected hotspots (*p = 0.1489*), nor is sample size (*p = 0.351*).

The recombination rate in hotspot regions for nine of the populations was on average between 22 and 236% higher than the average recombination rate of the genome (Table S4). The exception was Guianna, which only showed an approximately 1% increase in average recombination rate in hotspots regions when compared to that of the non-hotspot regions. For Guianna, we also compared the recombination rate inside hotspots to their surrounding regions (+/− 5 kb). We found that hotspot regions had a rate ~ 42% higher rate than their neighboring regions. This result leads us to believe that the 1% higher average recombination rate in the Guianna hotspots when compared to the entire genome may be due to an increased ability to detect hotspots in regions of low recombination for this population. Additionally, Guianna presents unusually large hotspots (average 8.6 kb, Table S4), which points to an especially low resolution in hotspot detection for this population.

Despite the majority of hotspots not being shared between populations, we conducted pairwise Fisher’s exact tests to verify whether there was significantly more hotspot overlap than expected (if hotspots were randomly distributed along the genome) between populations. For most pairs of populations, we found significantly more hotspot overlap than expected (Table 2). There were three comparisons that did not show significantly more overlap than expected: Amelonado-Nacional, Amelonado-Purus, and Criollo-Nacional. A Mantel test comparing distances between populations based on shared hotspots and F_ST_ values between populations resulted in a significant correlation between them (*r = 0.66*, *p = 0.002*).

**Table 2:** Fisher’s exact test *p*-values for pairwise comparisons of recombination hotspot locations between populations of *T. cacao*. We conducted 45 comparisons, corresponding to a Bonferroni correction cutoff value of *α = 0.0011*

Population	Ame	Con	Cri	Cur	Gui	Iqu	Mar	Nac	Nan
Amelonado	–	–	–	–	–	–	–	–	–
Contamana	<2e-07	–	–	–	–	–	–	–	–
Criollo	<9e-05	<5e-13	–	–	–	–	–	–	–
Curaray	<3e-05	<3e-37	<5e-08	–	–	–	–	–	–
Guianna	<3e-06	<1e-37	<7e-07	<4e-20	–	–	–	–	–
Iquitos	<4e-08	<6e-87	<2e-11	<3e-16	<2e-29	–	–	–	–
Maranon	<6e-13	<7e-77	<2e-11	<2e-20	<5e-33	<4e-64	–	–	–
Nacional	0.0015	<2e-43	0.0212	<7e-14	<3e-06	<6e-14	<3e-13	–	–
Nanay	0.0004	<2e-44	<9e-11	<4e-16	<2e-21	<3e-39	<2e-38	<9e-06	–
Purus	0.1782	<4e-117	<2e-05	<2e-29	<1e-33	<2e-39	<8e-43	<6e-27	<2e-21

To study the effects of demographic history more closely, shared hotspots were converted to dimensions of a multiple correspondence analysis and modeled along a previously constructed drift tree [35]. Modeling the dimension as a Brownian motion was a better fit (AIC = 79.4) than modeling it as an Ornstein-Uhlenbeck (OU) process (AIC = 81.4), which is consistent with the small number of hotspots shared between populations. The model assuming Brownian motion is consistent with pure drift driving differentiation of a trait along a genealogy, while an OU process is consistent with a higher trait maintenance (stabilizing selection).

### Identifying DNA sequence motifs associated with the locations of recombination hotspots

We used RepeatMasker to analyze the set of recombination hotspots that were present in at least eight *T. cacao* populations (17 total hotspots; referred to as ubiquitous hotspots), as well as the consensus set of recombination hotspots and the reference genome. In order to determine whether a particular set of DNA sequence repeats was overrepresented in ubiquitous hotspots, the percentage of DNA sequence that was identified as potentially being from retroelements or DNA transposon was compared to an empirical distribution. The percentage of observations from the distribution which were greater than the observed are reported in Table 3. While retroelements were found to be underrepresented in the ubiquitous hotspots, DNA transposons were marginally overrepresented.

**Table 3:** Percentage of DNA sequences identified as either retroelements or DNA transposons, and total interspersed repeats. Observed values for the entire *T. cacao* genome, for all recombination hotspots (HS), and ubiquitous hotspots (hotspots in the same location in at least eight different populations). Also presented are mean percentage of these sequences for 1000 simulations of hotspots equivalent in size and count as the ubiquitous set and the percentile at which the observed value for the ubiquitous set is found in the distribution of the simulated set (Sim)

Measures	Observed % ubiquitous HS	Observed % all HS	Observed % whole genome	Mean % Sim	% Sim > ubiquitous
Retroelements	2.34	9.45	11.12	11.11	99.9
DNA transposons	1.94	1.64	1.10	1.10	5.4
Total	4.28	11.09	12.21	12.22	99.7

### Identifying genomic features associated with the location of recombination hotspots

We found an overrepresentation of recombination hotspots at transcriptional start sites (TSSs) and transcriptional termination sites (TTSs) in all ten of the *T. cacao* populations (Table 4). The level of overrepresentation of hotstpots in particular regions was compared to a null expectation based on simulations of hotspots of the same size as the ones detected, distributed randomly along the chromosomes. For all populations, all 1000 simulations showed a lower proportion of overlap with TSSs and TTSs than the observed. In the case of exons and introns, seven populations (Contamana, Criollo, Iquitos, Maranon, Nacional, Nanay, Purus) had an observed value that was lower than all, or almost all (Purus for exons), simulations. Three of the remaining four populations (Amelonado, Curaray, and Nanay) had no clear trend in either direction (Table 4). The final population (Guianna) showed an overrepresentation of hotspots in both exons and introns.

**Table 4:** Proportion of simulated chromosomes that presented a lower number of hotspots intersecting with TSSs, TTSs, exons, and introns than the observed chromosomes. TSSs and TTSs are considered to be the 500 bp upstream and downstream of transcribed regions, respectively

Population	TSSs (500 bp)	TTSs (500 bp)	Exons	Introns
Amelonado	1	1	0.602	0.527
Contamana	1	1	0	0
Criollo	1	1	0	0
Curaray	1	1	0.346	0.058
Guianna	1	1	1	1
Iquitos	1	1	0	0
Maranon	1	1	0	0
Nacional	1	1	0	0
Nanay	1	1	0.027	0.237
Purus	1	1	0.004	0

## Discussion

The set of ten *T. cacao* populations, which includes wild, long-established and recently established populations as well as a domesticated population, has provided us a unique opportunity to study differences in recombination in populations of the same species with varying evolutionary histories [35, 46]. We also explored differences in the location of recombination hotspots between the populations and found that the conservation of hotspots between them generally mirrors their patterns of pairwise genetic differentiation. Additionally, we found that TSSs and TTSs are strongly associated with recombination hotspots in all populations, which is consistent with previous findings in plants [12, 15, 19]. This factor seems to play an important role in determining the location of novel hotspots. Finally, hotspots that are shared by at least eight populations appear to be associated with DNA transposons, pointing to a potential mechanism for the maintenance of recombination hotspots at the population-divergence timescale. Understanding how recombination rates vary between genetically differentiated populations of the same species is an important step toward disentangling the role of recombination in genetic differentiation.

### Higher recombination rate in domesticated cocoa population tied to mutations in FIGL-1 protein

We found that the eight long-established, wild *T. cacao* populations show an average recombination rate (*r*) within the range of recombination rates measured for other suporrosids (Table 1; 45). The average recombination rate for these *T. cacao*, populations, was 4.07 cM/Mb, which is very similar to that of *Juglans regia* (4.05 cM/Mb [47];), and comparable to other woody rosids (e.g. *Populus deltoides*, 8.13 cM/Mb [48]; *Mangifera indica*, 7.15 cM/Mb [49]; *Citrus clementina*, 3.6 cM/Mb [50]) [45]. This places *T. cacao* on the high end of known recombination rates for its order but comfortably in the range of other long-lived, woody plants. For all ten populations, the mean recombination rate was found to be greater than the median. This is consistent with high rate outlier values; an expected result in the presence of recombination hotspots. Despite recombination rates for eight of the ten populations being of the same order of magnitude, pairwise comparisons of average rates indicated that most populations have a significantly different rate of recombination from the others. The only exception were Nacional and Nanay whose average rates were not significantly different from each other. These two populations, however, are not more closely related to each other than they are to other populations, based on genetic differentiation [35]. We interpret this result as suggestive that their similarity is not due to genetic similarity, but some other factors, e.g. epigenetics.

Two *T. cacao* populations (Criollo, the domesticated variety and Guianna, the recently established wild population) show unusually high average recombination rates when compared to the eight long-established wild populations. Despite a small sample for some populations, including Criollo, we found no linear trend between sample size and recombination rate (Table S5). Additionally, the rates calculated for the two wild, small-sample populations (Curaray and Nacional) were consistent with those of other wild populations. This makes us confident in our estimates for the Criollo and Guianna populations. To further verify that our findings were consistent with those of similar studies we compared it to estimates of *Medicago truncatula* recombination made using the same method [12]. We used the effective population size for *M. truncatula* from Siol et al. (2007) to convert the estimate of ρ from Paape et al. (2012), to *r* (=433 cM/Mb) [12, 51]. This estimate of *r* for *M. truncatula* was comparable to the mean rate found for the Criollo population (Table 1) [12, 51]. One possible explanation for the higher recombination rate observed for the Criollo population is domestication; which has been observed to increase recombination rates, particularly in plants [39]. Previous work has shown genomic evidence of Criollo domestication, revealed by its much higher drift parameter when compared to other populations [35]. The high recombination rate observed in Guianna (46.5 cM/Mb) can be explained in a similar way; while Guianna does not show a strong signature of domestication, it is the most recently established population [46] and has undergone a recent bottleneck [35]. We hypothesize from this result that the Guianna population is undergoing the initial stages of domestication, and its increased recombination is an early indicator of this. Another possibility is that the high recombination rates estimated for Criollo and Guianna can be explained by biases in estimation caused by errors associated to small samples or low genetic variation. However, the recombination rates for Amelonado (another population with low variation) and Purus (a population with small sample size) did not present this problem.

Analyses exploring mutations of putative recombination suppression genes [29] could help disentangle the nature of this extreme variation in recombination rate in the Criollo and Guianna populations. It has been widely discussed that although actual recombination rates (chiasmata per bivalent) might increase in domestic plant populations, effective recombination is likely to be reduced in domestic populations as a result of increased LD and long runs of homozygosity. The interpretation for this observation is that it is likely that chromosomes might physically recombine at higher rates, but if homologous chromosomes contain the same sequence then no appreciable exchange of variants among different chromatid backgrounds can occur [37]. We find significantly higher estimates of median recombination rates for domesticated Criollo populations across the genome. Given previous research suggesting the key function of FIGL-1 on suppressing recombination rates in *Arabidopsis*, we investigated if mutations in this protein could explain the differences in recombination rate. Our results suggest that missense mutations in FIGL-1 may have impaired the activity of this protein in domesticated populations, reducing its efficacy as a recombination suppressor. The mutation showing the largest effect (KK_215) is close to the domain that has been previously shown to be involved in the interaction of FIGL-1 with RAD51 and DMC1 [41] and thus we hypothesize that FIGL-1 is in part responsible for the increase recombination rate in domesticated cacao populations. Further work is still needed to test this hypothesis, such as using yeast two-hybrid systems similar to the work performed by Fernandes et al. (2018a) to show whether the mutations interfere with the normal function of FIGL-1 [29]. These mutations were not found in increased frequency in Guianna, suggesting that the underlying mechanisms leading to increased recombination rates in this population are different to those in the Criollo population.

The likelihood of detecting hotspots of recombination in the genome can be affected by the amount of uncertainty in the estimates of recombination across sites or regions. Yet, we have been unable to identify any study where the magnitude of the uncertainty in the estimates of recombination are assessed to address this issue. We have performed careful comparisons and assessed the magnitude of the uncertainty in the estimation of recombination rates to show that this uncertainty is several orders of magnitude smaller than the variation in recombination rates across the genome (Table S1).

### Sharing of hotspots between populations correlates with genetic distance

Understanding the pattern and rate of change of recombination hotspots at the population level can elucidate their role in shaping genome architecture, impacting how effectively selection operates [1]. We found that a large proportion (55.5%) of hotspots detected are unique to a single population. The observed variability of hotspot location between populations points to demographic history not being the main driver of recombination hotspot location. However, the hotspots tend to appear in similar regions, as demonstrated by the Fisher’s exact tests (Table 2). This dichotomy can be explained by considering that the proportion of the genome occupied by recombination hotspots is very low, so even a small proportion of hotspots from two different populations being in the same region is enough for the Fisher’s exact test to recognize them as significantly similar. This small but significant similarity can occur by recombination being limited in its possible positioning along the genome, but not to the point of forcing hotspots to occur consistently in the same locations, and thus maintaining some level of stochasticity.

Given the significant proportion of overlapping hotspots between populations, it was still important to explore whether the similarities can be explained by shared genetic history. If demographic history explains the evolution of hotspot location, we would expect that more closely related populations would have a higher percent of overlapped hotspots. A significant relationship was found between population differentiation (F_ST_) and the distance between populations based on shared hotspots (Mantel test, *r = 0.66*, *p = 0.002*). This result indicates that, to some extent, the genetic differentiation and the location of hotspots are mirroring each other, which could be due to recombination hotspots being a product of the shared history between the populations. However, since recombination rates were estimated using a coalescent-based method, we expect historical relationships to be represented in our findings to some extent. We transformed the information of hotspot overlap to model hotspots as quantitative traits changing along a population tree [35]. Our results show that a Brownian motion model, which assumes that the trait is evolving following a random walk, is a better fit for the shared hotspot data than an Ornstein-Uhlenbeck model, which assumes that stabilizing selection is regressing the trait value to its mean, reducing variation over time. This suggests that drift alone could explain the evolution of the location of recombination hotspots. However, the absolute number of hotspots that are shared among populations indicates that demographic history is insufficient to explain the evolution of recombination hotspots in this species. In other organisms, recombination hotspots are frequently associated to specific genomic features (including TSSs and TTSs) [15, 18–20, 52] or DNA sequence motifs [14, 16, 21]. These factors can affect the landscape of recombination, contributing to the patterns of shared hotspot locations between populations that we observe in *T. cacao*.

While we do not detect all the hotspots in these populations and not all the hotspots detected are necessarily true positives, this proportion of unique hotspots can be seen as an indicator that the turnover rate for hotspots is faster than the time it took the 10 populations to differentiate. The detection rate for LDhot is approximately 55% under constant population conditions, and greater when a recent bottleneck has occurred [53, 54]. Only two of the populations in this study (Criollo and Guianna) have a known recent bottleneck [35]. However, Criollo is the only one of these two with an unusually low hotspot count (Table S4). Criollo’s low number of detected hotspots can be a product of its increased genome-wide recombination rate, making the signal of hotspots less pronounced. In a similar fashion, the increased overall recombination rate of Guianna may be affecting the detectability of hotspot regions, limiting our ability to resolve the limits of hotspots. It is important to note that our hotspots are unusually large for all populations (Table S4), which is likely a product of our low sample size leading to low resolution when resolving hotspot regions. Further work, increasing the sample size per population will contribute to increasing the resolution of these estimates.

Our ability to measure recombination in ten distinct populations allows us to analyze the relationship between population genetic processes and recombination. Our results suggest that the pattern of gains and losses of recombination hotspots is very dynamic and the landscape of recombination changes rapidly during the process of diversification within a species. This dynamism can have a tremendous impact on the adaptive dynamics of a species, and it should be taken into account, considering that theoretical studies tend to assume that recombination rates are constant during the evolution of populations [55, 56].

### Known retrotransposon sequences are underrepresented in hotspot regions

The analysis of 17 hotspots shared between at least eight populations of *T. cacao* found an underrepresentation of retroelements and a marginal overrepresentation of DNA transposons when compared to the entire genome (Table 3). These results are not entirely surprising as it has already been suggested that transposable elements (TEs) tend to be enriched in areas of low recombination in *Drosophila* as a consequence of selection against TE activity that could lead to chromosome instability [57]. However, the marginal over-representation of DNA transposons in the most conserved recombination hostspot is unexpected, given that all previous observations have shown a reduced representation of mobile elements in areas with high recombination rate [57]. It is possible that DNA transposons are at least partly responsible for the maintenance of recombination hotspots as populations diverge, from which we expect that site-directed recombination is more frequent in these locations of the genome. However, the low percentage of these sequences observed in the set of all hotspots (Table 3) indicates that these sequences only have a small effect on the maintenance of hotspots. It has been observed in humans that short DNA motifs enriched for repeat sequences determine the location of 40% of hotspots enriched for recurrent non-allelic homologous recombination [58]. One potential explanation for why natural selection does not eliminate hotspots in these regions is the possibility that these regions do not produce a large enough mutational load for natural selection to remove them from the population [58].

### Hotspots overrepresented near transcription start and stop sites

For all ten populations, an overrepresentation of hotspots was found in the areas immediately preceding and following transcribed regions of the chromosome. This matches the findings of previous studies in *Arabidopsis thaliana* [19], *Taenipygia guttata* and *Poephila acuticauda* [20], and humans [18]. The most likely explanation is that recombination events within genes are selected against. The rationale being that a recombinant chromosome that undergoes a double-strand break in the middle of a coding region will have a higher risk of being inviable, and therefore not represented in the current set of chromosomes for its population. Recombination occurring in transcription start and stop sites, on the other hand, does a much better job at breaking up haplotypes or shuffling alleles in different genomic backgrounds, while preserving the functionality of coding regions. This rationale is supported by previous findings of increased recombination rates in these regions [19]. It is also supported by results from PRDM9 knock-out *Mus musculus*, which has shown a reversion to hotspots located near TSSs [59]. The enrichment of *T. cacao* hotspots in TSSs and TTSs is thus a reasonable result given that zinc-finger binding motifs and potential modifiers like PRDM9 have not been identified in this species.

## Conclusions

Our results show a pattern where recombination rates in the eight long established, wild populations of *T. cacao* are of a similar magnitude to each other and to other plants, but they show a high diversity in location and number of hotspots of recombination. This diversity cannot be explained solely by the process of diversification of the populations. One potential hypothesis for the rapid turnover of hotspots of recombination and the relative differences in recombination among populations is that epigenetic changes are involved in controlling the turnover of recombination in plants. This hypothesis is not unreasonable given the recent observation of epigenetic control of recombination in plants [60]. Further theoretical and simulation work should be done in order to better understand the implications of the rapidly changing recombination hotspots in adaptive dynamics.

We also show an overall underrepresentation of hotspots in exons and introns for most populations, which is consistent with purifying selection acting against changes that could result in disruptions of gene function. On the other hand, we observed an overrepresentation of hotspots in TTSs and TSSs for all ten populations. This could impact the maintenance and spread of beneficial traits in the population by shuffling allelic variants of genes without disrupting their function. We hypothesize that the enrichment of hotspots of recombination in TTSs and TSSs can have an important impact in the spread of beneficial mutations across different genomic backgrounds; increasing the rate of adaptation to selective pressures (e.g. selection for improved pathogen response).

We found that the recombination rates for the domesticated population of cacao (Criollo) had an unusually high recombination rate. Domestication has been found to increase recombination rates in plants [9]. Additionally, the FIGL-1 protein plays an important role in repressing recombination rates in *Arabidopsis thaliana* [41]. We found that the increased recombination rates in Criollo could be explained in part by mutations to FIGL-1 orthologs in *T. cacao*. This finding provides evidence for a potential mechanism for increased recombination rates in domesticated plants.

## Methods

### Estimating recombination rates using LDhat

Sequence data were downloaded from the Cacao Genome Database and NCBI (Accession PRJNA486011), including the reference sequence for each chromosome and the full genome annotation (*Theobroma cacao* cv. Matina 1–6 v1.1) [61]. Processing was done using the pipeline from [35] available at the github repository https://github.com/oeco28/Cacao_Genomics. Full genome data was used from a total of 73 individuals (146 chromosomes) across 10 populations (35). We filtered single nucleotide polymorphism data and excluded rare variants (minor allele frequency < = 0.05) per population. Separate variant files per population per chromosome were then phased using default conditions with SHAPEIT2 [62] under default parameters. Haplotype files were converted back to phased variant calling format (vcf) for its downstream analysis. We have also phased the data with Beagle [63], using a burn-in of 10,000 iterations, and estimations done over 10,000 iterations. No appreciable differences were observed between the two methods and Beagle phasing was maintained for the analyses. The reason for performing the phasing separately for each population is that linkage disequilibrium patterns are expected to be affected by population structure. The ten populations have been shown to be unique clusters with very little admixture between them [35], and the individuals used in this study were those whose ancestry was clearly from a single population. VCFTools [64] was used to remove all singletons and doubletons (i.e. SNPs where the minor allele count is less than three). Only bi-allelic single nucleotide polymorphisms (SNPs) were retained and were exported in LDhat format. The sample size and post-filtering SNP count for each population can be found in Table S5.

In order to estimate recombination rates, we used the *interval* routine from LDhat [42], a program that implements coalescent resampling methods to estimate historical recombination rates from SNP data. To reduce computation time, each chromosome was split into windows, each containing 2000 SNPs. To counteract the overestimation of recombination rate produced at the ends of the windows, an overlap of 500 SNPs was left between consecutive windows. The final window for each chromosome did not always match the general scheme, so the final 2000 SNPs were taken (making the overlap with the second to last window variable, but never less than 500 SNPs) (Fig. S4). Once these windows were generated, LDhat was run over each window using 100 million iterations, sampling every 10,000 iterations (10,000 total points sampled), with a block penalty of 5. Lookup tables with a grid of 100 points, population mutation rate parameters (θ) of 0.1 and 0.001, and a number of sequences (n) of 50 were used for all populations. Downstream analyses were conducted using the results from the θ = 0.1 runs of LDhat. We used the same θ for all populations since estimates from [35] ranged from π = 0.27% to π = 0.37%, all comfortably within an order of magnitude of each other. The first 50 million iterations were discarded as burn-in. Once recombination rates were calculated, 250 positions were cut off from both windows involved in each overlap, so that the estimates for the first half of the overlap was taken from the end of the preceding window and the estimates for the second half of the overlap were taken from the beginning of the following window. The final overlap in each chromosome was split in order to remove 250 SNPs from the second to last window, regardless of the remaining size of the last window. The remaining rate estimates were then merged in order to obtain recombination rates for the entire chromosome. This was done for each chromosome of each population.

The estimation of recombination rates with LDhat is approximated using a sampling scheme with a Markov Chain Monte Carlo (MCMC) algorithm as implemented in the *interval* routine. The inference of recombination rates is the result of the integration of estimated parameter values across iterations with the routine *stats*. In the majority of recent studies where LDhat or LDhelmet are used [12, 14, 16, 18–21, 52], whether there is convergence of the Markov chains has not been explicitly investigated. One study that we are aware of has used simulations to assess whether their small sample size affected their ability to obtain reliable estimates of recombination using LDhelmet [65] but did not assess the uncertainty of the estimates from the MCMC process itself. We argue that evaluation of convergence is important to assess the confidence in the estimated reported values, especially if there is interest in analyzing the differences in recombination rate along the genome. Visual inspection of pilot runs of the analysis demonstrated that convergence was not achieved after running 40 M iterations, which is why the length of the chains was increased to 100 M iterations. Additionally, we explored the uncertainty in the estimates of recombination site-wise by integrating over the trace of the estimates for recombination rate to infer the 95% Credibility Interval. We then estimated the 95% interval of recombination estimates range across all sites in the genome to have an overall measure of uncertainty that we compared to the median 95% Credibility Interval for the trace of each position.

### Comparing recombination rates between populations

In order to compare recombination rates, the effective population size (*N*_*e*_) calculated for each population [35] was used to convert rates in *N*_*e*_*r* to *r*. We tested the difference between the median whole-genome rate of recombination (*r*) between populations first by conducting a Kruskal-Wallis test, and later comparing pairs of populations using the Wilcoxon rank-sum test. We used the kruskal.test and wilcox.test function from the stats package in R) [66]. There were 46 comparisons, making the Bonferroni correction cutoff value: α = 0.0011.

Furthermore, to explore whether trends in population-level mean and median recombination rates could be explained with possible confounding variables, we used linear regressions of the form:

$$ y={\beta}_0+x{\beta}_j+{\varepsilon}_j $$

Where *y* is a vector of the mean or median recombination rate for the ten populations, *β*_0_ is the intersect, *x* is a vector of some predictor variable, and *β*_*j*_ is the effect of the predictor variable on recombination rate. The variables we tested were mean N_e_, inbreeding coefficient (F, from [35]), and sample size. We ran these linear models using the lm function from the stats package in R.

### Modeling the effect of recombination suppressing genes

Suppressors of recombination identified in Arabidopsis and other systems, FIGL-1 and FLIP, have not yet been identified in *T. cacao* [61]. We performed a reciprocal BLAST search using a newly generated databased containing sequences of FIGL-1 and FLIP obtained from ncbi [67–69]. After identification of orthologous copies of FIGL-1 and FLIP, we extracted the annotated variants responsible for missense mutations in the proteins from data generated in Cornejo et al. (2018) [35]. We then used these mutations to estimate the frequency of homozygous genotypes with alternative alleles (different to the reference). Because the reference genome belongs to the Amelonado populations, the population that presents the lowest estimated median recombination rate in our work, we used the frequency of homozygous alternatives to infer the impact of missense mutations under the assumption of a recessive model on the recombination rate. For this, we estimated the homozygosity for missense mutations and eliminated those found in complete correlation (r^2^ = 1) and fitted a generalized linear model of the form:

$$ y={\beta}_0+{\beta}_j+{\varepsilon}_j. $$

Where *y* is a vector of the median recombination rate across populations, *β*_*0*_ is the intersect (in this case the Amelonado median recombination rate) and *β*_*j*_ is the effect of the homozygosity in position *j*. We assumed a Gaussian link function for the model.

### Determining hotspot regions using LDhot

Recombination hotspots were estimated with LDhot [42], a likelihood-based program that tests whether a single distribution model or a two-distribution model better explains the observed recombination rates in 1 kb sliding windows (default), for each chromosome. Each chromosome was run in its entirety, with the number of simulations (nsims) set to 1000. The resulting potential hotspots were refined by an α of 0.001, and overlapping hotspots were merged. This method therefore detects hotspots by comparing rates in 1 kb windows to the rates in the surrounding regions.

To determine the set of consensus hotspots, the hotspots from all populations were merged. Two hotspots from different populations were considered to be shared if they both overlapped with the same hotspot in the consensus set. To summarize all shared hotspots, a Boolean matrix was constructed, in which a population having a hotspot that overlaps with a hotspot in the consensus list leads to an indication of presence of the consensus hotspot in that population. This matrix was used to determine hotspots shared by two or more populations.

### Comparing hotspot location between populations

A Fisher’s exact test was run for each pair of populations in order to determine whether hotspots for the pair of populations overlap significantly more than expected. The BED files containing the location of the recombination hotspots for each pair of populations were compared using Bedtools:fisher [70]. The number of comparisons was 45, making the Bonferroni correction cutoff value: α = 0.0011.

In order to compare the relationships between populations based on shared hotspots we calculated Jaccard distances (distance function, philentropy package, R) [71] and compared them to a published F_ST_ matrix (Table S6) [35] using a Mantel test (mantel.rtest function, ade4 package, R) [72–75]. The F_ST_ estimates from [35] were generated using Weir and Cockerham’s estimator [76].

In order to model the presence or absence of hotspots along a drift tree, a multiple correspondence analysis was used on the Boolean matrix of shared hotspots using the MCA function from the FactoMineR package in R [77]. Nine dimensions were retained and used as traits along a previously generated drift tree [35]. Using the Rphylopars package in R [78], the dimensions were modeled as Brownian motion (assuming a random walk, where the variance of a trait increases over time) and as an Ornstein-Uhlenbeck process (assuming that stabilizing selection regresses the trait value to the mean, limiting the changes in variance over time). The fit of the two models were compared using the AIC values for the best fitting models of each type.

Additionally, to explore whether the number of recombination hotspots detected for a population could be explained with possible confounding variables we used linear regressions of the form:

$$ y={\beta}_0+x{\beta}_j+{\varepsilon}_j $$

Where *y* is a vector of the of hotspot count for all ten populations, *β*_0_ is the intersect, *x* is a vector of some predictor variable, and *β*_*j*_ is the effect of the predictor variable on the number of hotspots. We ran these linear regressions using the lm function from the stats package in R, and we tested mean N_e_ and sample size.

### Identifying DNA sequence motifs associated with the locations of recombination hotspots

Motifs associated with hotspots were found using RepeatMasker [79]. The entire genome, the set of consensus hotspots, and a set of ubiquitous hotspots (hotspots shared by at least eight of the populations) were examined with RepeatMasker, using normal speed and “*Theobroma cacao*” in the species option. In order to determine whether ubiquitous hotspots were enriched for particular DNA sequences, a set of the same number and size of sequences was randomly selected from the genome using Bedtools:shuffle [70] and examined with RepeatMasker. This simulation was repeated one thousand times and a null distribution against which observed values were compared was constructed from the results.

### Identifying genomic features associated with the location of recombination hotspots

Testing whether recombination hotspots were overrepresented near particular genomic features was done by using a resampling scheme to establish null expectations and then comparing the observed value to the empirical distribution. For each feature, locations were retrieved and the number of observed hotspots that overlap with this feature were counted. To determine whether this number of overlapping hotspots was unusually high or low, a set of hotspots that matched the number of hotspots and the size of each hotspot was simulated. These simulated hotspots were placed randomly along the chromosome, using a uniform distribution. The simulation was run 1000 times and the number of simulated hotspots that overlap with the true genomic features was measured for each simulation. The simulations generate an expected distribution of overlap with the genomic feature, and the true value was then compared to the distribution. When simulated hotspots overlapped, the location of one of them was sampled again. Features tested were: Transcriptional start sites (TSSs), transcriptional termination sites (TTSs), exons, and introns. TSSs and TTSs are considered to be the 500 bp upstream and downstream of coding regions respectively.

The reason for the proposed novel resampling scheme is that, if the size and distribution of genomic features and hotspots were not taken into account, it would set unrealistic expectations for the overlap between features under a null model of no association. In this sense, the null model would be inappropriate and potentially inflate the false positive rate.

## Supplementary information

**Additional file 1: Table S1.** The median of the upper and lower bounds of the 95% Credibility Interval for the trace of estimates of *r* (in cM/Mb) from all positions in the genome are presented for each population (i.e. Position L95 and Position U95). The upper and lower bounds of the 95% probability interval for the median estimate of *r* for each population is also presented (i.e. Genome L95 and Genome U95). The quotients of the upper and lower bounds for each of the two intervals point to a much larger genome-wide variation in *r* than per-position variation in the trace for the estimate of *r*. **Table S2.** Mean and median genome-wide recombination rates (*r*) in cM/Mb for all ten *T. cacao* populations obtained using LDhat with θ = 0.001. **Table S3.** Name of *T. cacao* gene coding for FIGL-1 and FLIP and amino acid mutations for FIGL-1 and FLIP orthologs. **Table S4.** Average hotspot size (in kb) and count for hotspots detected in each population and average for all populations. The quotient of the average recombination rate within hotspots and the average genome-wide recombination rate is reported for each population. **Table S5.** Sample size and post-filtering SNP count for all ten populations of *Theobroma cacao* for which recombination maps were generated. The proportion of the genome that is callable is also reported. We also include the geographic location of the population and whether it is a domesticated variety. **Table S6.** Pairwise F_ST_ values for the ten populations of *Theobroma cacao.* Values from Cornejo et al. (2018) [35]. **Figure S1.** Drift tree constructed using treemix [80] for the 10 *T. cacao* populations. Distances between populations are based on the drift parameter. Modified from Cornejo et al. (2018) [35]. **Figure S2.** Distribution of *log*_*10*_ recombination rates (*log*_*10*_*(r)*) along the genomes of the ten *T. cacao* populations. The sample size (N) is reported for each population. **Figure S3**. The left panel shows the frequency of individuals that are homozygous for the alternative allele of amino acid mutations in a *T. cacao* FLIP ortholog. Alternative allele is defined in terms of the Amelonado reference genome. The right panel shows the *log*_*e*_ transformed recombination rates (*r*). The populations are in the same order in both panels. **Figure S4.** Example of the window layout for a 10,750 SNP chromosome. The 2000 SNP long windows are represented by alternating horizontal and vertical lines and the overlaps between them are represented by square crosshatches. Braces above the chromosome indicate the regions from which recombination rates are extracted to generate the chromosome-wide recombination rates.

## Data Availability

Rate and summary files from LDhat runs as well as hotspots for each population along with scripts for LDhat and LDhot runs, the resampling schemes used, and additional analyses is available in the following github repository: https://github.com/ejschwarzkopf/recombination-map.

## Abbreviations

TSSs: Transcriptional start sites
TTSs: Transcriptional termination sites
LD: Linkage disequilibrium
TEs: Transposable elements
SNPs: Single nucleotide polymorphisms
r: Recombination rate
N_e_: Effective population size
4N_e_r: Effective recombination rate
OU: Ornstein-Uhlenbeck
