# Supplementary material for: Genetic differentiation and intrinsic genomic features explain variation in recombination hotspots among cocoa tree populations
Source: BMC Genomics. 2020 Apr 29;21:332. doi: 10.1186/s12864-020-6746-2 (PMC7191684; doi:10.1186/s12864-020-6746-2)
Supplement: Supplementary file 1 — Additional file 1: Table S1. The median of the upper and lower bounds of the 95% Credibility Interval for the trace of estimates of r (in cM/Mb) from all positions in the genome are presented for each population (i.e. Position L95 and Position U95). The upper and lower bounds of the 95% probability interval for the median estimate of r for each population is also presented (i.e. Genome L95 and Genome U95). The quotients of the upper and lower bounds for each of the two intervals point to a much larger genome-wide variation in r than per-position variation in the trace for the estimate of r. Table S2. Mean and median genome-wide recombination rates (r) in cM/Mb for all ten T. cacao populations obtained using LDhat with θ = 0.001. Table S3. Name of T. cacao gene coding for FIGL-1 and FLIP and amino acid mutations for FIGL-1 and FLIP orthologs. Table S4. Average hotspot size (in kb) and count for hotspots detected in each population and average for all populations. The quotient of the average recombination rate within hotspots and the average genome-wide recombination rate is reported for each population. Table S5. Sample size and post-filtering SNP count for all ten populations of Theobroma cacao for which recombination maps were generated. The proportion of the genome that is callable is also reported. We also include the geographic location of the population and whether it is a domesticated variety. Table S6. Pairwise FST values for the ten populations of Theobroma cacao. Values from Cornejo et al. (2018) [35]. Figure S1. Drift tree constructed using treemix [80] for the 10 T. cacao populations. Distances between populations are based on the drift parameter. Modified from Cornejo et al. (2018) [35]. Figure S2. Distribution of log10 recombination rates (log10(r)) along the genomes of the ten T. cacao populations. The sample size (N) is reported for each population. Figure S3. The left panel shows the frequency of individuals that are homozygous for the alternative al [file 12864_2020_6746_MOESM1_ESM.docx]

**Supplementary Materials:**

Genetic differentiation and intrinsic genomic features explain variation in recombination hotspots among cocoa tree populations

Enrique J. Schwarzkopf, Juan C. Motamayor, Omar E. Cornejo (ocornejo@gmail.com)

**Tables:**

**Table S1.** The median of the upper and lower bounds of the 95% Credibility Interval for the trace of estimates of *r* (in cM/Mb) from all positions in the genome are presented for each population (i.e. Position L95 and Position U95). The upper and lower bounds of the 95% probability interval for the median estimate of *r* for each population is also presented (i.e. Genome L95 and Genome U95). The quotients of the upper and lower bounds for each of the two intervals point to a much larger genome-wide variation in *r* than per-position variation in the trace for the estimate of *r*.

| Population | Position  L95 | Position  U95 | Genome  L95 | Genome  U95 | Position Range Quotient | Genome Range Quotient |
| --- | --- | --- | --- | --- | --- | --- |
| Amelonado | 6.35e-05 | 7.67e-03 | 2.33e-05 | 31.3 | 120.75 | 1.34e+06 |
| Contamana | 1.63e-01 | 1.34 | 1.40e-04 | 26.4 | 8.22 | 1.88e+05 |
| Criollo | 87.5 | 43.1 | 5.35e-02 | 1,660 | 4.92 | 3.11e+04 |
| Curaray | 5.15e-01 | 2.63 | 2.72e-04 | 20.2 | 5.11 | 7.40e+04 |
| Guianna | 9.76e-01 | 12.6 | 1.81e-04 | 296 | 12.90 | 1.63e+06 |
| Iquitos | 5.50e-05 | 6.52e-03 | 1.58e-05 | 24.5 | 186.29 | 1.55e+06 |
| Maranon | 1.98e-04 | 7.40e-02 | 2.31e-05 | 35.2 | 373.35 | 1.52e+06 |
| Nacional | 4.80e-05 | 6.50e-03 | 2.76e-05 | 36.6 | 135.60 | 1.33e+06 |
| Nanay | 1.65e-04 | 3.06e-02 | 2.06e-05 | 35.2 | 185.32 | 1.71e+06 |
| Purus | 3.98e-03 | 5.24e-01 | 2.00e-04 | 63.5 | 131.87 | 3.18e+05 |

**Table S2.** Mean and median genome-wide recombination rates (*r*) in cM/Mb for all ten *T. cacao* populations obtained using LDhat with θ= 0.001.

| Population | Mean *r* (cM/Mb) | Median *r* (cM/Mb) |
| --- | --- | --- |
| Amelonado | 4.04 | 4.78e-03 |
| Contamana | 4.55 | 9.75e-01 |
| Criollo | 715.35 | 668.90 |
| Curaray | 5.85 | 3.12 |
| Guianna | 63.24 | 4.34 |
| Iquitos | 2.68 | 7.95e-03 |
| Maranon | 4.16 | 3.38e-02 |
| Nacional | 6.00 | 1.32e-01 |
| Nanay | 5.43 | 1.61e-01 |
| Purus | 11.27 | 1.96 |

**Table S3.** Name of *T. cacao* gene coding for FIGL-1 and FLIP and amino acid mutations for FIGL-1 and FLIP orthologs.

| **Protein** | **FIGL-1** | **FLIP** |
| --- | --- | --- |
| **Gene** | *Thecc1EG017182t2* | *Thecc1EG020410t1* |
| **Mutations** | K9Q, F66L, D114Y, T155I, D189V, K195M, E215K, S235N, R270Q, A285S, S291T, V548I, G566S, A567V, M580T | L102F, S153T, K174T, Y140N, V125A, H203R, Q237K, V406L, S430Y, R446Q, M497I, L521M, D696V, L729I, M739I, A746G, K875Q, P906L |

**Table S4.** Average hotspot size (in kb) and count for hotspots detected in each population and average for all populations. The quotient of the average recombination rate within hotspots and the average genome-wide recombination rate is reported for each population.

| Population | Mean hotspot size (kb) | Hotspot count | Hotspot *r* / Genome *r* |
| --- | --- | --- | --- |
| Amelonado | 6.9 | 1325 | 3.36 |
| Contamana | 6.1 | 5184 | 2.25 |
| Criollo | 6.1 | 887 | 1.22 |
| Curaray | 5.8 | 2303 | 1.45 |
| Guianna | 8.6 | 3655 | 1.01 |
| Iquitos | 7.0 | 3258 | 2.18 |
| Maranon | 6.8 | 3296 | 2.32 |
| Nacional | 6.9 | 2202 | 1.77 |
| Nanay | 7.6 | 3818 | 1.76 |
| Purus | 6.3 | 3972 | 1.76 |
| Average | 6.9 | 2989.9 | 1.91 |

**Table S5.** Sample size and post-filtering SNP count for all ten populations of *Theobroma cacao* for which recombination maps were generated. The proportion of the genome that is callable is also reported. We also include the geographic location of the population and whether it is a domesticated variety.

| Population | Sample size  (chromosome) | SNP count | Proportion callable | Longitude | Latitude | Domesticated |
| --- | --- | --- | --- | --- | --- | --- |
| Amelonado | 11 (22) | 373,789 | 0.68 | -43 | -12 | No |
| Contamana | 9 (18) | 2,097,618 | 0.87 | -75.08 | -7.5 | No |
| Criollo | 4 (8) | 309,818 | 0.69 | -89.4 | 18.81 | Yes |
| Curaray | 5 (10) | 1,106,871 | 0.70 | -76.88 | -1.955 | No |
| Guianna | 9 (18) | 770,729 | 0.915 | -55.71 | 1.29 | Not clear |
| Iquitos | 7 (14) | 1,575,711 | 0.922 | -73 | -3.5 | No |
| Maranon | 14 (28) | 1,783,226 | 0.955 | -75.15 | -4.65 | No |
| Nacional | 4 (8) | 718,099 | 0.89 | -73.83 | -5.12 | No |
| Nanay | 10 (20) | 830,885 | 0.94 | -75.55 | -0.73 | No |
| Purus | 6 (12) | 1,184,181 | 0.9 | -68.25 | -9.2 | No |

**Table S6.** Pairwise F_ST_ values for the ten populations of *Theobroma cacao.* Values from Cornejo et al. (2018) [35].

| Population | Ame | Con | Cri | Cur | Gui | Iqu | Mar | Nac | Nan |
| --- | --- | --- | --- | --- | --- | --- | --- | --- | --- |
| Amelonado | 0 | - | - | - | - | - | - | - | - |
| Contamana | 0.279 | 0 | - | - | - | - | - | - | - |
| Criollo | 0.646 | 0.267 | 0 | - | - | - | - | - | - |
| Curaray | 0.375 | 0.169 | 0.341 | 0 | - | - | - | - | - |
| Guianna | 0.362 | 0.257 | 0.579 | 0.362 | 0 | - | - | - | - |
| Iquitos | 0.280 | 0.181 | 0.391 | 0.225 | 0.310 | 0 | - | - | - |
| Maranon | 0.217 | 0.218 | 0.388 | 0.269 | 0.197 | 0.222 | 0 | - | - |
| Nacional | 0.414 | 0.162 | 0.441 | 0.200 | 0.405 | 0.225 | 0.280 | 0 | - |
| Nanay | 0.217 | 0.243 | 0.488 | 0.316 | 0.325 | 0.190 | 0.222 | 0.343 | 0 |
| Purus | 0.324 | 0.165 | 0.374 | 0.221 | 0.308 | 0.191 | 0.215 | 0.235 | 0.255 |

**Figures:**

**Figure S1.** Drift tree constructed using treemix [80] for the 10 *T. cacao* populations. Distances between populations are based on the drift parameter. Modified from Cornejo et al. (2018) [35].

**Figure S2.** Distribution of *log_10_* recombination rates (*log_10_(r)*) along the genomes of the ten *T. cacao* populations. The sample size (N) is reported for each population.

**Figure S3**. The left panel shows the frequency of individuals that are homozygous for the alternative allele of amino acid mutations in a *T. cacao* FLIP ortholog. Alternative allele is defined in terms of the Amelonado reference genome. The right panel shows the *log_e_* transformed recombination rates (*r*). The populations are in the same order in both panels.

**Figure S4.** Example of the window layout for a 10,750 SNP chromosome. The 2,000 SNP long windows are represented by alternating horizontal and vertical lines and the overlaps between them are represented by square crosshatches. Braces above the chromosome indicate the regions from which recombination rates are extracted to generate the chromosome-wide recombination rates.
